# Clinical use of artificial intelligence in endometriosis: a scoping review

**DOI:** 10.1038/s41746-022-00638-1

**Published:** 2022-08-04

**Authors:** Brintha Sivajohan, Mohamed Elgendi, Carlo Menon, Catherine Allaire, Paul Yong, Mohamed A. Bedaiwy

**Affiliations:** 1 Schulich School of Medicine & Dentistry, Western University, London, ON Canada; 2 Department of Obstetrics and Gynecology, Faculty of Medicine, University of British Columbia, Vancouver, BC Canada; 3 Biomedical and Mobile Health Technology Laboratory, Department of Health Sciences and Technology, ETH Zurich, Zurich, Switzerland; 4 British Columbia Women’s Hospital, Vancouver, BC Canada

**Keywords:** Diagnostic markers, Predictive markers, Reproductive disorders

## Abstract

Endometriosis is a chronic, debilitating, gynecologic condition with a non-specific clinical presentation. Globally, patients can experience diagnostic delays of ~6 to 12 years, which significantly hinders adequate management and places a significant financial burden on patients and the healthcare system. Through artificial intelligence (AI), it is possible to create models that can extract data patterns to act as inputs for developing interventions with predictive and diagnostic accuracies that are superior to conventional methods and current tools used in standards of care. This literature review explored the use of AI methods to address different clinical problems in endometriosis. Approximately 1309 unique records were found across four databases; among those, 36 studies met the inclusion criteria. Studies were eligible if they involved an AI approach or model to explore endometriosis pathology, diagnostics, prediction, or management and if they reported evaluation metrics (sensitivity and specificity) after validating their models. Only articles accessible in English were included in this review. Logistic regression was the most popular machine learning method, followed by decision tree algorithms, random forest, and support vector machines. Approximately 44.4% (*n* = 16) of the studies analyzed the predictive capabilities of AI approaches in patients with endometriosis, while 47.2% (*n* = 17) explored diagnostic capabilities, and 8.33% (*n* = 3) used AI to improve disease understanding. Models were built using different data types, including biomarkers, clinical variables, metabolite spectra, genetic variables, imaging data, mixed methods, and lesion characteristics. Regardless of the AI-based endometriosis application (either diagnostic or predictive), pooled sensitivities ranged from 81.7 to 96.7%, and pooled specificities ranged between 70.7 and 91.6%. Overall, AI models displayed good diagnostic and predictive capacity in detecting endometriosis using simple classification scenarios (i.e., differentiating between cases and controls), showing promising directions for AI in assessing endometriosis in the near future. This timely review highlighted an emerging area of interest in endometriosis and AI. It also provided recommendations for future research in this field to improve the reproducibility of results and comparability between models, and further test the capacity of these models to enhance diagnosis, prediction, and management in endometriosis patients.

## Introduction

Endometriosis is a chronic, gynecologic condition^1^ estimated to affect 190 million women worldwide^2^. This benign, but often debilitating condition is thought to impact ~10% of women based on extrapolations of pelvic pain and subfertility in the general population^3^ and of those that are symptomatic, the prevalence is thought to be 30% to 50%^4^. True prevalence rates are difficult to estimate because this condition is often underreported, undiagnosed or misdiagnosed^1^. In Canada, the national societal burden of endometriosis is estimated at CAD $1.8 billion annually based on treatment costs, caregiver costs, quality of life and work absenteeism^5^. Endometriosis poses a large economic and disease burden on society and the precise scope of the problem remains unknown.

Endometriosis is characterized by extrauterine growth of endometrial-like tissue in areas of the pelvis (i.e., ovaries), bowels, bladder, and peritoneum^6^. These growths are rarely found in the thoracic region, and other organ systems^7,8^. Endometriosis has three predominant phenotypes: superficial endometriosis, endometriomas and deep endometriosis (DE)^8,9^. There are many staging systems for endometriosis, including the American Society for Reproductive Medicine classification system: stage I (minimal), stage II (mild), stage III (moderate), and stage IV (severe)^10,11^. However, given the complexity of this disease, it is difficult to universally stage and characterize under the present systems. Significant research has been done in recent years in attempts to elucidate the pathogenesis of this disease and many etiological factors are currently being explored including immune-mediated, inflammatory, genetic and environmental components^12,13^.

The signs and symptoms of this disease are non-specific and can vary in severity, creating clinical heterogeneity, which adds to the diagnostic difficulty associated with this disease^8^. Patients can present with a range of symptomatology depending on the type of endometriosis, location of implants, stage, and severity including but not limited to dysmenorrhea, dyspareunia, abdominal pain, chronic pelvic pain, menorrhagia, bowel symptoms, urinary symptoms, and subfertility or infertility^8^. Due to the combination of non-specific symptoms, a long differential list, lack of provider awareness, unnecessary investigations, and a lack of non-invasive diagnostic tools, many patients experience significant delays in receiving an endometriosis diagnosis^1,14–16^. The current literature has documented diagnostic delays of up to 6 to 12 years globally before patients receive a definitive diagnosis and adequate management^1,17,18^. Currently, the gold standard diagnostic procedure for endometriosis remains laparoscopic visualization of lesions followed by histologic confirmation of ectopic endometriotic implants^8^, a costly and invasive process that requires a skilled clinician. Transvaginal ultrasonography is a commonly used clinical technique in endometriosis screening and diagnosis, given its non-invasive nature and widespread accessibility^8^.

In the past 5 years, the emergence of artificial intelligence (AI) has spread rapidly into healthcare; it has demonstrated marked potential in disease diagnostics, treatments, and a higher-level analysis of large biomedical datasets^19,20^. With the increase in digitization in healthcare, AI presents novel opportunities to decrease the amount of time required for diagnosis and to streamline care in many settings^19^. Machine learning (ML) is a subset of AI and includes common methods such as logistic regression with the use of training and test sets and support vector machines (SVMs)^19^. Currently, AI has been used to analyze multi-omics, clinical, behavioral/wellness, environmental and research and developmental data^19^, and it has been applied to decision-making, patient self-management, triage, understanding disease mechanisms, and drug discovery^21,22^. However, AI methods require an expert’s oversight to help inform the model’s development since clinical problems are often complex and multifaceted^19^. Additionally, the privacy and the security of patient data remain a consideration when introducing new technology into healthcare; thus researchers should be aware of any risks associated with AI models^19^.

**Fig. 1:**
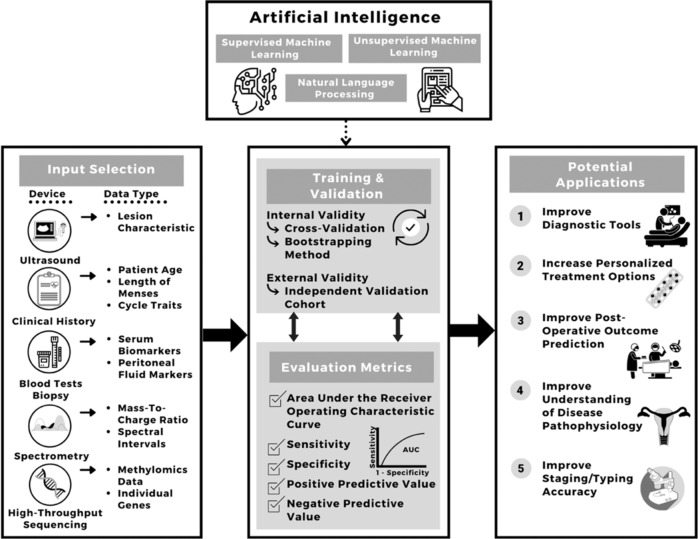
Potential area of use for artificial intelligence applications in endometriosis. This figure was created by B.S. and M.E.

From fetal heart monitoring to reproductive medicine, AI technologies have been used in the field of obstetrics and gynecology and have demonstrated the potential to significantly aid in prediction of outcomes^22–25^. Given the diversity of its use in the clinical context, there is great potential to apply AI to the complex challenges presented by endometriosis and improve non-invasive diagnostics to reduce the delays and human error associated with diagnosis^22^. However, clinicians face significant challenges in the field of AI applications including a widespread lack of understanding about different AI methods and the competencies and limitations of AI technologies^21^. This review examines the different ways AI methods have been applied to solve pressing issues in endometriosis diagnostics, prediction, and research as shown in Fig. 1. By providing a thorough understanding of the different models and their application to clinical problems, and by analyzing their strengths and limitations, recommendations will be provided to help future researchers adequately develop AI models to advance the field of endometriosis.

## Results

### Study selection

**Fig. 2:**
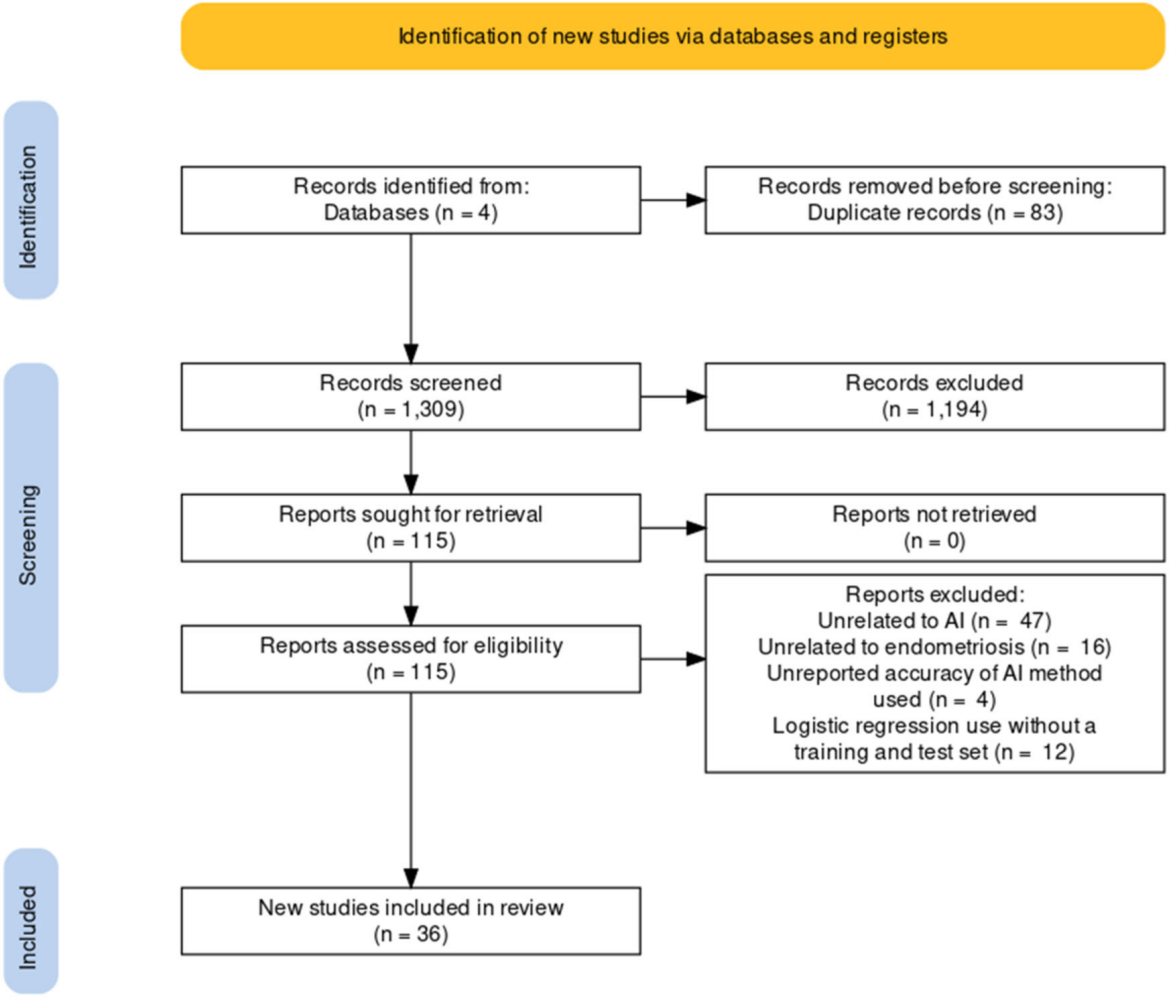
Workflow of the study. Flowchart of study identification, inclusion, and exclusion criteria.

A total of 1309 titles were identified by searching the PubMed, Medline-OVID, EMBASE, and CINAHL database, and 115 full-texts were eligible for screening after studies were excluded during the title and abstract-screening stages. Of these, 79 papers were excluded in the final review based on our exclusion criteria and 36 studies were included in the final review (Fig. 2). A summary of the eligible studies and extracted study characteristics is shown in Table 1. The majority of studies were predominantly retrospective designs (*n* = 20) using data from large clinical databases and registries and some prospective designs (*n* = 16); no randomized studies were included. Samples sizes ranged from modest numbers of 26 patients with endometriosis^26^ to 1396 symptomatic patients^27^, with the average sample size being 245 individuals for studies exploring diagnosis and prediction in endometriosis.

**Table 1:** Description of the studies.

Year	Author [ref.]	Study design	Intervention	Purpose	Objective	Sample size	AI accuracy for best model
2022	Bendifallah et al.^50^	Retrospective	Logistic Regression, Random Forest, Decision Tree, eXtreme Gradient Boosting, Voting Classifier (soft/hard)	Prediction	Predict likelihood of endometriosis based on 16 essential clinical and symptom-based features related to patient history, demographics, endometriosis phenotype and treatment	1126 endometriosis patients, 608 controls	SE = 93% SP = 92%
2022	Bendifallah et al.^35^	Prospective	Logistic Regression, Random Forest eXtreme Gradient Boosting, AdaBoost	Diagnosis	Diagnosis of endometriosis using a blood-based mRNA diagnostic signature	200 plasma samples (153 cases, 47 controls)	SE = 96.8% SP = 100%
2021	Maicus et al.^61^	Prospective	Resnet (2 + 1)D	Diagnosis	Classification of the state of the Pouch of Douglas using the sliding sign test on ultrasound	749 transvaginal ultrasound videos (414 training set, 139 validation set, 196 test set)	SE = 88.6% SP = 90%
2021	Guerriero et al.^59^	Retrospective	K-Nearest Neighbor, Naïve Bayes, Neural Networks, SVM, Decision Tree, Random Forest, Logistic Regression	Prediction	Detection of endometriotic bowel involvement in rectosigmoid deep endometriosis	333 patients	SE = 72% SP = 73%
2021	Li et al.^52^	Retrospective	Deep Machine Learning Algorithm (NNET)	Diagnosis	Diagnosis of endometriosis based on genes	213 patients	SE = 100% SP = 61.1%
2020	Matta et al.^30^	Retrospective Case–Control	Logistic Regression, ANN, SVM, Adaptive Boosting, PLSDA	Research	Identify biomarkers of internal exposure in adipose tissue most associated with endometriosis	99 women (44 controls, 55 cases)	SE = NR SP = NR
2020	Akter et al.^53^	Retrospective	New Ensemble Machine Learning Classifier (GenomeForest)	Diagnosis	Classifying endometriosis versus control patients using RNAse and enrichment-based DNA-methylation datasets	38 single-end RNA-sequence samples, 80 MBD-sequence DNA-methylation samples	Transcriptomics Data SE = 93.8% SP = 100% Methylomics Data SE = 92.9% SP = 88.6%
2020	Perrotta et al.^54^	Prospective Observational Cross-Sectional Pilot	Random Forest-Based Machine Learning Classification Analysis	Diagnosis	Diagnosis of endometriosis using gut and/or vaginal microbiome profiles	59 women (24 controls, 35 endometriosis patients)	SE = NR SP = NR
2020	Guo et al.^58^	Retrospective Cohort	Logistic Regression	Prediction	Predict any-stage and stage 3/4 endometriosis before surgery in infertile women	1016 patients (443 without endometriosis, 377 patients with stage 1/2 endometriosis, 196 patients with stage 3/4 endometriosis)	SE = NR SP = NR
2021	Vesale et al.^45^	Retrospective	Logistic Regression	Prediction	Predict likelihood of voiding dysfunction after surgery for deep endometriosis	789 patients	SE = NR SP = NR
2019	Benoit et al.^46^	Retrospective	Logistic Regression	Prediction	Predict likelihood of a live birth after surgery followed by ART for patients with endometriosis-related infertility	297 women	SE = NR SP = NR
2019	Lee et al.^29^	Retrospective	Recommendation System	Research	Identify diseases associated with endometriosis	1,730,562 controls, 11,273 cases	SE = NR SP = NR
2019	Braga et al.^36^	Prospective Case–Control	PLSDA	Diagnosis	Develop an adjuvant tool for diagnosis of grades 3 and 4 endometriosis in infertile patients	50 endometriosis serum samples, 50 control samples	SE = NR SP = NR
2019	Chattot et al.^57^	Prospective Observational	Logistic Regression	Prediction	Predict rectosigmoid involvement in endometriosis using preoperative score	119 women undergoing surgery for endometriosis	SE = NR SP = NR
2019	Knific et al.^31^	Retrospective	Decision Tree, Linear Model, K-Nearest Neighbor, Random Forest	Diagnosis	Diagnosis of endometriosis based on plasma levels of proteins and patients’ clinical data	210 patients	SE = 40% SP = 65%
2019	Parlatan et al.^37^	Retrospective	K-Nearest Neighbor, SVM, PCA	Diagnosis	Diagnosis of endometriosis using non-invasive Raman spectroscopy-based classification model	94 serum samples (49 endometriosis, 45 controls)	SE = 89.7% SP = 80.5%
2019	Akter et al.^55^	Retrospective	Decision Tree, PLSDA, SVM, Random Forest	Diagnosis	Classify endometriosis versus control biopsy samples using transcriptomics or methylomics data	38 samples in transcriptomics dataset, 77 samples in methylomics dataset	Transcriptomics Data SE = 81.3% SP = 95.5% Methylomics Data SE = 76.2% SP = 80%
2018	Bouaziz et al.^28^	Retrospective	NLP	Research	Using NLP to extract data by text mining of the endometriosis-related genes in the PubMed database	724 genes retrieved	SE = NR SP = NR
2017	Dominguez et al.^33^	Prospective Case–Control	SVM	Diagnosis	Diagnosis of endometriosis using lipidomic profiling of endometrial fluid in patients with ovarian endometriosis	12 endometriosis, 23 controls	SE = 58.3% SP = 100%
2016	Ghazi et al.^38^	Prospective Cohort	PLSDA, Multi-Layer Feed Forward ANN, QDA	Prediction	Determine classifier metabolites for early prediction risk of disease	31 infertile women with endometriosis, 15 controls	SE = NR SP = NR
2015	Reid et al.^60^	Prospective Observational	Logistic Regression	Prediction	Use mathematical ultrasound models to determine whether a combination of transvaginal sonography markers could improve prediction of Pouch of Douglas obliteration	189 women with suspected endometriosis	Model 1 SE = 88% SP = 97% Model 2 SE = 88% SP = 99%
2014	Lafay Pillet et al.^47^	Prospective	Logistic Regression	Diagnosis	Diagnose DE before surgery for patients operated on for endometriomas	164 patients with DIE, 162 with no DIE	SE = 51% SP = 94%
2014	Tamaresis et al.^56^	Retrospective	Margin Tree Classification	Diagnosis	Detect and stage pelvic endometriosis using genomic data from endometrium	148 endometrial samples	SE = NR SP = NR
2014	Wang et al.^39^	Prospective Case–Control	Genetic Algorithm, Decision Tree Algorithm, Quick Classifier Algorithm	Diagnosis	Diagnosis of endometriosis and stage using peptide profiling	122 patients	SE = 90.9% SP = 92.9%
2013	Wang et al.^51^	Retrospective	Decision Tree	Prediction	Predict medical care decision rules for patients with recurrent pelvic cyst after surgical interventions	178 case records	SE = NR SP = NR
2012	Ballester et al.^48^	Prospective Longitudinal Study	Logistic Regression	Prediction	Prediction of clinical pregnancy rate in patients with endometriosis	142 infertile patients with DIE	SE = 66.7% SP = 95.7%
2012	Fassbender et al.^40^	Retrospective	LSSVM	Diagnosis	Diagnosis of endometriosis undetectable by ultrasonography	254 plasma samples (89 controls, 165 endometriosis patients)	SE = 88% SP = 84%
2012	Fassbender et al.^41^	Retrospective	LSSVM	Diagnosis	Diagnosis of endometriosis through mRNA expression profiles in luteal phase endometrium biopsies	49 endometrial biopsies	SE = 91% SP = 80%
2012	Vodolazkaia et al.^34^	Retrospective Cohort	Logistic Regression, LSSVM	Diagnosis	Diagnosis of endometriosis in symptomatic patients without U/S evidence of endometriosis	121 controls, 232 endometriosis patients	SE = 81% SP = 81%
2012	Dutta et al.^42^	Prospective	PLSDA	Prediction	Identification of predictive biomarkers in serum for early diagnosis of endometriosis in a minimally invasive manner	22 endometriosis, 23 controls	SE = 81.8% SP = 91.3%
2012	Nnoaham et al.^27^	Prospective Observational	Logistic Regression	Prediction	Predict any-stage endometriosis and stage 3 and 4 disease with a symptom-based model	1396 symptomatic women	SE = 82.6% SP = 75.8%
2010	Wang et al.^26^	Retrospective	ANN	Prediction	Screening for biomarkers of eutopic endometrium in endometriosis patients	26 patients	SE = 91.7% SP = 90.9%
2009	Wolfler et al.^43^	Prospective Exploratory Cohort	Genetic Algorithm	Prediction	Predict endometriosis before laparoscopy using patterns of serum proteins in symptomatic patients	91 symptomatic patients	SE = 81.3% SP = 60.3%
2009	Stegmann et al.^62^	Prospective Cohort	Logistic Regression	Prediction	Prediction of lesions that have high probability of containing histologically-confirmed endometriosis	114 women with complete data on 487 lesions	SE = 88.4% SP = 24.6%
2008	Wang et al.^44^	Retrospective	ANN	Diagnosis	Diagnostic model to correctly detect endometriosis and no endometriosis in serum samples using potential biomarkers of endometriosis	66 serum samples	SE = 91.7% SP = 90%
2005	Chapron et al.^49^	Prospective	Logistic Regression	Prediction	Predict presence of posterior deep endometriosis among women with chronic pelvic pain symptoms	134 women scheduled for laparoscopy for chronic pelvic pain symptoms	SE = 68.6% SP = 77.1%

*NR* not reported, *PLSDA* partial least squares discriminant analysis, *QDA* quadratic discriminant analysis, *SVMs* support vector machines, *ANNs* artificial neural networks, *LSSVMs* least squares support vector machines, *PCA* principal component analysis, *NLP* natural language processing, *DE* deep endometriosis, *U/S* ultrasound, *miRNAs* microRNAs, *ART* assisted reproductive technology, *RNA* ribonucleic acid, *DNA* deoxyribonucleic acid, *MBD* methyl binding domain*, SE* sensitivity, *SP* specificity.

### Study characteristics

In the field of endometriosis, AI utilization spanned three overarching categories: predicting outcomes in endometriosis populations, building diagnostic models, and improving research efficacy. Most interventions were developed to assist with prediction of endometriosis in patients. However, the type, stage and specific characteristics of endometriosis that these interventions predicted, differed among the studies, depending on the research question generated by the authors. Approximately 44.4% (*n* = 16) of the studies analyzed the predictive capabilities of AI approaches in patients with endometriosis, while 47.2% (*n* = 17) explored diagnostic capabilities. The predictive capabilities differed between studies but included many aims such as predicting fertility therapy success in endometriosis patients, the likelihood of endometriosis versus other pelvic pain pathologies, predicting the presence of DE, and many more as seen in Table 1. Only 8.33% (*n* = 3) of the studies used AI technologies to advance the understanding of disease pathophysiology^28–30^. The AI methods that were used included: logistic regression, K-nearest neighbor, Naïve Bayes, random forest, decision tree, SVMs, neural networks, classification tree analysis, genetic algorithm, least squares support vector machines (LSSVMs), partial least squares discriminant analysis (PLSDA), margin tree classification, quick classifier algorithm, quadratic discriminant analysis (QDA), natural language processing (NLP), principle component analysis (PCA), adaptive boosting, eXtreme gradient boosting, voting classifier (hard/soft), deep learning and new ensemble ML classifiers. However, logistic regression (*n* = 15) was the AI intervention that was most frequently used to build predictive and diagnostic models.

The types of inputs used in different AI models varied among the studies. Four studies used biomarkers as the specific inputs for their final predictive model, but the types of biomarkers differed including: angiogenic factors, cytokines, serum microRNAs signatures, and other metabolite biomarkers. Some studies also used metabolite spectra as inputs for their AI models (*n* = 10) however, there was significant diversity between the type of spectrometry method (i.e., Raman spectrometry versus hydrogen nuclear magnetic resonance [1H-NMR] Carr-Purcell-Meiboom-Gill [CPMG] spectrometry) and the specific mass-dependent velocity (m/z, mass divided by charge number) peak ranges that were used among the studies. Other studies also used genetic variables such as large transcriptomics datasets (*n* = 5) and clinical factors (*n* = 6) as inputs for their final models. The clinical factors that were used in different models demonstrated some similarity with age, history of pelvic surgery, dysmenorrhea, and pelvic pain being commonly used variables. However, many studies used different combinations, thresholds and classifiers for these variables in their models. For instance, various combinations of severe dysmenorrhea, primary dysmenorrhea, and secondary dysmenorrhea were used in different ML models.

Although the AI approaches were heterogenous, most models generally achieved sensitivity and specificity above 85%, as demonstrated in Table 1. All of the studies (*n* = 33) used a validation process to train and validate AI models with various methods of cross-validation (i.e., bootstrapping method, leave-one-out cross-validation, etc.) or by implementing a validation/test cohort not used in the initial training set. Table 1 also reports on sensitivity and specificity for the models.

Given the heterogeneity in the purpose of the AI intervention, type and stage of endometriosis being examined, type of AI methodology used, and evaluation metrics, the included studies were grouped into six categories based on the inputs used to create the AI models. These categories are discussed in detail below.

### Diagnostic or predictive models for endometriosis using biomarkers

Four different studies^31–35^ examined the use of biomarkers as inputs to create diagnostic or predictive AI models in endometriosis populations. As seen in Table 2, the type of biomarkers used differed among the studies. Knific et al.^31^ was the only study that used protein ratios while others used metabolites^33^, miRNAs^35^ and other biomarkers^34^. Knific et al.^31^ and Bendifallah et al.^35^ were the only studies in this category to use the random-forest method to develop a diagnostic model for endometriosis and the accuracy of Knific et al.’s^31^ model was reported to be 59%^31^ —the lowest accuracy for all the models in this category—while the clinical accuracy of Bendifallah et al.’s^35^ model was significantly higher with a sensitivity and specificity of 96.8 and 100%. One study used LSSVMs^34^ and the accuracy of this method was deemed to be 79% with a sensitivity and specificity of 82% and 75%, respectively. One study also used SVMs to develop a diagnostic model for endometriosis using lipidomic profiling of endometrial fluid in patients with ovarian endometriosis^33^. The accuracy of this method was reported to be 85.7% with a sensitivity and specificity of 58.3% and 100%, respectively. It should be noted that among the four studies that were examined, there were no commonalities in the specific biomarker inputs used; thus, it is difficult to compare the accuracy of each AI model given the differences in the inputs used. The pooled SE and SP for each study’s most accurate model were 85.6% and 85%, respectively^33–35^.

**Table 2:** Diagnostic and predictive moels built using biomarkers.

AI methods used	Authors [ref.]	Stage of endometriosis	Type of endometriosis	Sample size	Inputs used	Method accuracy
Random Forest	Bendifallah et al.^35^	rASRM Class I–II and Class III–IV	Not specified	200 patients (153 endometriosis, 47 controls)	86 miRNAs composing a diagnostic blood signature	SE = 96.8% SP = 100%
	Knific et al.^31^	All four stages of endometriosis^a^	Not specified	210 patients (116 endometriosis, 94 controls)	Proteins ratios for the following: CTACK/MCP-3, MCP-3/CTACK, CCL11/I-309, X6Ckine/MCP-1, CTACK/SCYB16, Gro-alpha/CTACK	SE = NR SP = NR
Logistic Regression	Bendifallah et al.^35^	rASRM Class I–II and Class III–IV	Not specified	200 patients (153 endometriosis, 47 controls)	86 miRNAs composing a diagnostic blood signature	SE = 96.8% SP = 100%
	Vodolazkaia et al.^34^	Not specified	U/S negative endometriosis	353 EDTA samples (232 endometriosis, 121 controls)	VEGF, Annexin V, CA-125, glycodelin, sICAM-1	SE = 82% SP = 75%
eXtreme Gradient Boost	Bendifallah et al.^35^	rASRM Class I–II and Class III–IV	Not specified	200 patients (153 endometriosis, 47 controls)	86 miRNAs composing a diagnostic blood signature	SE = 90.3% SP = 100%
AdaBoost	Bendifallah et al.^35^	rASRM Class I–II and Class III–IV	Not specified	200 patients (153 endometriosis, 47 controls)	86 miRNAs composing a diagnostic blood signature	SE = 96.8% SP = 100%
Support Vector Machines	Dominguez et al.^33^	Not specified	Ovarian endometriosis	35 patients (12 endometriosis, 23 controls)	123 differentially expressed metabolites in endometrial fluid	SE = 58.3% SP = 100%
Least Squares Support Vector Machines	Vodolazkaia et al.^34^	Not specified	U/S negative endometriosis	353 EDTA samples (232 endometriosis, 121 controls)	VEGF, Annexin V, CA-125, sICAM-1	SE = 82% SP = 75%

*rASRM* revised American Society of Reproductive Medicine, *NR* not reported, *U/S* ultrasound, *CTACK* cutaneous T cell-attracting chemokine, *MCP-3* monocyte chemotactic protein 3, *CCL-11* C-C motif chemokine ligand 11, *I-309* chemokine ligand 1, *X6Ckine* C-C motif chemokine 21, *MCP-1* monocyte chemoattractant protein 1, *SCYB16* chemokine ligand 16, *Gro-alpha* growth regulated oncogene-alpha, *VEGF* vascular endothelial growth factor, *CA-125* cancer antigen 125, *sICAM-1* soluble intercellular adhesion molecule-1, *SE* sensitivity, *SP* specificity.

^a^Minimal, mild, moderate and severe stages of endometriosis were included.

### Diagnostic or predictive models for endometriosis using protein spectra

Ten studies^26,36–44^ used various metabolite spectra as their primary inputs to develop diagnostic and predictive models in endometriosis populations. In this specific problem formulation, it is important to note the methodology that is used. The most popular method to determine metabolite spectra for model development was surface-enhanced laser desorption/ionization time-of-flight mass spectrometry, which was used by four studies^26,41,43,44^. The pooled SE for the models with highest accuracy in each study was 91.7%, while the pooled SP was 81.1%^26,37–44^. Table 3 presents the other methods of spectrometry and spectroscopy that were used to determine the metabolite spectra of interest for the model inputs.

**Table 3:** Diagnostic and predictive models built using protein spectra.

AI methods used	Authors [ref.]	Spectrometry or spectroscopy method	Stage of endometriosis	Type of endometriosis	Sample size	Inputs used	Method accuracy
Support Vector Machines	Parlatan et al.^37^	Raman Spectroscopy	All four stages of endometriosis^a^	Not specified	94 serum samples (49 endometriosis, 45 controls)	790–1729 cm^−1^ spectral interval	SE = 87.5% SP = 100%
k-nearest neighbor (weighted)	Parlatan et al.^37^	Raman Spectroscopy	All four stages of endometriosis^a^	Not specified	94 serum samples (49 endometriosis, 45 controls)	790–1729 cm^−1^ spectral interval	SE 100% SP = 100%
Partial least squares discriminant analysis (PLSDA)	Braga et al.^36^	Mass Spectrometry	Stage 3 and 4	Not specified	100 patients (50 endometriosis, 50 controls)	Positive ionization m/z = 758.7234, 786.7585, 758.7155, 782.7239, 369.4541; negative ionization *m*/*z* = 279.3316, 215.1182, 255.3261, 281.3487, 283.36375	SE = NR SP = NR
	Dutta et al.^42^	1H-NMR Spectroscopy	Stage 1 and 2	Not specified	45 patients (22 endometriosis, 23 controls)	TSP, lipoproteins (LDL and VLDL), unsaturated lipid, creatinine, L-Arginine, glucoerophosphatidylcholine, D-glucose, ornithine, citrate, L-lysine, tyrosine, L-histidine, L-phenylalanine, formate, choline, L-threonine, acetate, L-glutamine, succinate, acetone, adipic acid, L-isoleucine, alanine, L-aspartate, 3-hydroxybutyric acid, propylene glycol, valine, leucine, creatine, pyruvate, lactate, 2-hydroxybutyrate	SE = 81.8% SP = 91.3%
Quadratic discriminant analysis	Ghazi et al.^38^	Nuclear magnetic resonance spectroscopy	Stage 2 and 3	Not specified	45 patients (31 endometriosis, 15 controls)	Chemical shift for all spectra between 0 to 5.5ppm	SE = NR SP = NR
Genetic algorithm	Wang et al.^39^	Liquid chromatography tandem mass spectrometry	All four stages of endometriosis^a^	Not specified	122 patients (60 endometriosis, 62 without endometriosis)	*m*/*z* = 1433.9, 1599.4, 2085.6, 6798, 3217.2	SE = 90.9% SP = 92.9%
	Wolfler et al.^43^	Surface-enhanced laser desorption/ionization time-of-flight mass spectrometry	Not specified	Not specified	91 symptomatic patients	Mass peaks between 2000 and 20000 Da	SE = 55.6% SP = 64.9%
Decision tree algorithm	Wang et al.^39^	Liquid chromatography tandem mass spectrometry	All four stages of endometriosis^a^	Not specified	122 patients (60 endometriosis, 62 without endometriosis)	36 differentially expressed peptide spectra	SE = 90% SP = 80.6%
	Wolfler et al.^43^	Surface-enhanced laser desorption/ionization time-of-flight mass spectrometry	Not specified	Not specified	91 symptomatic patients	Mass peaks between 2000 and 20000 Da	SE = 92.7% SP = 62.8%
Quick classifier algorithm	Wang et al.^39^	Liquid chromatography tandem mass spectrometry	All four stages of endometriosis^a^	Not specified	122 patients (60 endometriosis, 62 without endometriosis)	36 differentially expressed peptide spectra	SE = 73.3% SP = 77.4%
Least squares support vector machines	Fassbender et al.^40^	Matrix-assisted laser desorption ionization time-of-flight mass spectrometry	Stage 1/2, stage 3/4	U/S negative endometriosis	254 plasma samples (165 endometriosis, 89 without endometriosis)	Minimal to mild endometriosis *m*/*z* = 4898, 5715, 8328, 9926, 14.698; moderate to severe endometriosis *m*/*z* = 3192, 4519, 2189, 4373, 7457; ultrasonography-negative endometriosis *m*/*z* = 2.058, 2456, 3.883, 14.694, 42.065	Minimal to mild endometriosis: SE = 75% SP = 86% Moderate to severe endometriosis: SE = 98% SP = 81% Ultrasonography-negative endometriosis: SE = 88% SP = 84%
	Fassbender et al.^41^	Proteomic surface-enhanced laser desorption ionization time-of-flight mass spectrometry	All four stages of endometriosis^a^	Not specified	49 endometrial biopsies (31 endometriosis, 18 without endometriosis)	*m*/*z* = 2072, 2973, 3623, 3680, 21113	SE = 91% SP = 80%
Artificial neural networks	Ghazi et al.^38^	Nuclear magnetic resonance spectroscopy	Stage 2 and 3	Not specified	45 patients (31 endometriosis, 15 controls)	Chemical shift for all spectra between 0 and 5.5ppm	SE = 50% SP = 17%
	Wang et al.^26^	Surface-enhanced laser desorption/ionization time-of-flight mass spectrometry	All four stages of endometriosis^a^	Not specified	39 patients (26 endometriosis, 13 controls)	*m*/*z* = 6898, 5891, 5385, 6448, 5425	SE = 91.7% SP = 90.9%
	Wang et al.^44^	Surface-enhanced laser desorption/ionization time-of-flight mass spectrometry	All four stages of endometriosis^a^	Not specified	66 serum samples (36 endometriosis, 30 controls)	*m*/*z* = 8142, 5640, 5847, 8940, 3269	SE = 91.7% SP = 90%

*NR* not reported, *m/z* mass-to-charge ratio, *ppm* parts per million, *Da* Dalton, *TSP* thrombospondin, *VLDL* very-low-density lipoprotein, *LDL* low-density lipoprotein, *1H-NMR* hydrogen-1 nuclear magnetic resonance, *U/S* ultrasound, *SE* sensitivity, *SP* specificity.

^a^Minimal, mild, moderate and severe stages of endometriosis were included.

Among the studies in this category, artificial neural networks (ANNs) were the most popular method used in three of the models^26,38,44^. However, although these three studies used the same type of AI intervention, the inputs varied greatly between them. Two studies used PLSDA to compute their final models^36,42^, albeit using different methodologies (mass spectroscopy^36^ and 1H-NMR spectrophotometer^42^). While the inputs also varied between both models, they both had a similar correct classification rates of 84%^36^ and 86.67%^42^. Further studies between similar inputs are needed to determine if PLSDA is an appropriate AI intervention to compute diagnostic and predictive models in endometriosis populations.

### Diagnostic or predictive models for endometriosis using clinical variables and symptoms

Six studies^45–50^ grouped in this category strongly preferred using logistic regression; two studies^50,51^ used decision tree methods to build a model and one study^50^ also used random forest, eXtreme gradient boosting and voting classifier (soft/hard) ML algorithms as shown in Table 4. Interestingly many studies in this category examined predictive and diagnostic model capabilities in patients with some form of deep endometriosis (*n* = 5). The pooled SE for the models with highest accuracy in each study was 81.7% while the pooled SP was 91.6%^47–50^. Specific inputs into each model varied as seen in previous categories with Bendifallah et al.^50^ using the largest number of clinical features for their models. However, there were some commonalities in the types of inputs that were used in each model. Patient age was the most frequently used input (*n* = 5) in diagnostic and predictive models using clinical variables. Given that endometriosis most commonly presents in reproductive-aged women, it is not surprising that age is the most frequent input in a diagnostic/predictive AI model. Other significant inputs included the presence or severity of dysmenorrhea, presence or severity of dyspareunia, visual analogic scale for dyspareunia, infertility, and previous surgery for endometriosis or pelvic surgery. Among the studies that did report SE and SP metrics, the SE values ranged from 51% to 95% and SP values ranged from 77.1 to 95.7%^47–50^.

**Table 4:** Diagnostic and predictive models built using clinical variables and symptoms.

AI methods used	Authors [ref.]	Stage of endometriosis	Type of endometriosis	Sample size	Inputs used	Method accuracy
Logistic Regression	Bendifallah et al.^50^	Not specified	Ovarian, superficial or deep endometriosis	Training set (1126 patients), validation set (100 patients)	Mother/daughter history of endometriosis, history of surgery for endometriosis, age, BMI, dysmenorrhea/VAS of dysmenorrhea, abdominal pain outside menstruation, pain suggesting of sciatica, pain during sexual intercourse, lower back pain outside menstruation, painful defecation, urinary pain during menstruation, right shoulder pain near or during menstruation, blood in the stools during menstruation, blood in urine during menstruation, absenteeism duration in the last 6 months, number of non-hormonal pain treatments used	SE = 95% SP = 81%
	Vesale et al.^45^	Not specified	Deep endometriosis with colorectal involvement	Training set (789 patients), validation set (333 patients)	Age, type of colorectal management, colpectomy and parametrectomy	SE = NR SP = NR
	Benoit et al.^46^	All four stages of endometriosis^a^	Not specified	297 patients who underwent ART after surgery for endometriosis-associated infertility	Age, duration of infertility, number of ICSI-IVF cycles, ovarian reserve, rAFS score	SE = NR SP = NR
	Lafay Pillet et al.^47^	Not specified	Deep endometriosis in patients with ovarian endometrioma	326 patients (164 with DE lesions associated with endometrioma, 162 patients with no associated DE lesions)	VAS of gastrointestinal symptoms ≥5 or of deep dyspareunia >5, duration of pain greater than 24 months, severe dysmenorrhea (defined as the prescription of the OCP for the treatment of a primary dysmenorrhea or the worsening of a secondary dysmenorrhea), primary or secondary infertility	SE = 51% SP = 94%
	Ballester et al.^48^	Not specified	Deep endometriosis	training set: 94 patients who underwent ICSI-IVF, validation set: 48 consecutive patients	Patient’s age, presence of DIE, AMH serum level >1 ng/ml, number of ICS-IVF cycles	SE = 66.7% SP = 95.7%
	Chapron et al.^49^	Not specified	Posterior deep endometriosis	134 patients (51 with posterior DE, 83 with other disorders)	Painful defecation during menses, VAS for dyspareunia > or =8, previous surgery for endometriosis, pain other than non-cyclic	SE = 68.6% SP = 77.1%
Decision Tree	Bendifallah et al.^50^	Not specified	Ovarian, superficial or deep endometriosis	Training set (1126 patients), validation set (100 patients)	See above.	SE = 91% SP = 66%
	Wang et al.^51^	Not specified	Ovarian endometriomas	178 case records	Patients’ basic information (age, number of pregnancies, number of births, number of miscarriages, past histories, menstruation periods, regularity of menstruations, periods of menstrual flow, severity of dysmenorrhea, urges to defecate, dyspareunia, whether other pains exist and other concomitant histories); clinical test values (endometrioma counts, sizes of endometriomas, follicle counts, CA125 blood values, sizes of uteruses, level of ovarian adhesions and contents of endometriomas); treatment-related information (medication prior to surgery, medication following surgery, route of drug administration, surgical method, surgical routine, UGA method, UGA site, UGA with irrigation and medication used)	SE = NR SP = NR
Random Forest	Bendifallah et al.^50^	Not specified	Ovarian, superficial or deep endometriosis	Training set (1126 patients), validation set (100 patients)	See above.	SE = 92% SP = 92%
eXtreme Gradient Boosting	Bendifallah et al.^50^	Not specified	Ovarian, superficial or deep endometriosis	Training set (1126 patients), validation set (100 patients)	See above.	SE = 93% SP = 92%
Voting Classifier (soft/hard)	Bendifallah et al.^50^	Not specified	Ovarian, superficial or deep endometriosis	Training set (1126 patients), validation set (100 patients)	See above.	Voting Classifier Soft SE = 93% SP = 88% Voting Classifier Hard SE = 91% SP = 92%

*NR* not reported, *DE* deep endometriosis, *ICSI-IVF* intracytoplasmic sperm injection in vitro fertilization, *rAFS* revised American Fertility Society, *OCP* oral contraceptive pill, *VAS* visual analogic scale, *BMI* body mass index, *CA-125* cancer antigen 125, *UGA* ultrasound-guided aspiration, *SE* sensitivity, *SP* specificity.

^a^Minimal, mild, moderate and severe stages of endometriosis were included.

### Diagnostic or predictive models for endometriosis using genetic variables

Models that were built using genetic variables as their primary inputs used a significantly larger number of inputs than any of the other six input categories referenced in this review. Only five studies^52–56^ used genetic variables to build their predictive and diagnostic models, however, the type of input varied between individual gene candidates^52,56^, large protein-coding gene datasets from transcriptomics and methylomics data^53,55^, and 16S rRNA gene amplicon data^54^. The AI methods used in this category included: deep ML algorithm, decision tree, GenomeForest (a new ensemble ML classifier), random-forest-based ML classification analysis, PLSDA, SVM, random forest, and margin tree classification. The pooled SE for the models with highest accuracy in each study was 96.7%, while the pooled SP was 70.7%^52,53,55^.

Two studies compared the use of large transcriptomics and methylomics datasets to build different AI models that were compared with each other^53,55^. As seen in Table 5, regardless of which AI method was used, the models built using the transcriptomics dataset outperformed the models built with the methylomics dataset, albeit marginally. Akter^53^ used GenomeForest, a novel ensemble technique based on chromosomal partitioning, to classify endometriosis and control samples using both transcriptomics and methylomics datasets. The authors concluded that this new classifier could help identify candidate biomarkers for endometriosis; they further demonstrated that three different ML models (GenomeForest, decision tree, and Biosigner) independently identified NOTCH3 as candidate gene with differential expression in the endometriosis samples^53,55^. ML methods may be of particular use when analyzing very large genomic datasets to help identify candidate genes that have altered expression in endometriosis patients versus control samples.

**Table 5:** Diagnostic and predictive models built using genetic variables.

AI methods used	Authors [ref.]	Stage of endometriosis	Type of endometriosis	Sample size	Inputs used	Method accuracy
Deep Machine Learning Algorithm	Li et al.^52^	All four stages of endometriosis^a^	Not specified	213 patients (142 endometriosis, 71 controls)	SCAF11, KIF3A, KRAS, MDM2	SE = 100% SP = 61.1%
GenomeForest	Akter et al.^53^	All four stages of endometriosis^a^	Not specified	Transcriptomics dataset: 16 endometriosis, 22 controls; methylomics dataset: 44 endometriosis, 36 controls	Genes in transcriptomics data and genomic regions in methylated data. 11 687 protein-coding genes (14 154 genes total)	For transcriptomics data: SE = 93.8% SP = 100% For methylomics data: SE = 92.9% SP = 88.6%
Random-Forest-based Machine Learning Classification Analysis	Perrotta et al.^54^	All four stages of endometriosis^a^	Not specified	59 patients (35 endometriosis, 24 controls)	Operational taxonomic unit and community state types in vaginal microbiome	SE = NR SP = NR
Decision Tree	Akter et al.^55^	All four stages of endometriosis^a^	Not specified	Transcriptomics dataset: 38 samples (16 endometriosis, 22 controls); methylomics dataset: 77 samples (42 endometriosis, 35 controls)	Transcriptomics: 14 154 genes; methylomics: 2 577 382 methylated regions	For transcriptomics: SE = 81.3% SP = 95.5% For methylomics: SE = 76.2% SP = 80%
Partial Least Squares Discrimination Analysis	Akter et al.^55^	All four stages of endometriosis^a^	Not specified	Transcriptomics dataset: 38 samples (16 endometriosis, 22 controls); methylomics dataset: 77 samples (42 endometriosis, 35 controls)	Transcriptomics: 14 154 genes; methylomics: 2 577 382 methylated regions	For transcriptomics: SE = 86.4% SP = 56.3% For methylomics: SE = 60% SP = 76.2%
Support Vector Machines	Akter et al.^55^	All four stages of endometriosis^a^	Not specified	Transcriptomics dataset: 38 samples (16 endometriosis, 22 controls); methylomics dataset: 77 samples (42 endometriosis, 35 controls)	Transcriptomics: 14 154 genes; methylomics: 2 577 382 methylated regions	For transcriptomics: SE = 63.6% SP = 43.8% For methylomics: SE = 40% SP = 61.9%
Random Forest	Akter et al.^55^	All four stages of endometriosis^a^	Not specified	Transcriptomics dataset: 38 samples (16 endometriosis, 22 controls); methylomics dataset: 77 samples (42 endometriosis, 35 controls)	Transcriptomics: 14 154 genes; methylomics: 2 577 382 methylated regions	For transcriptomics: SE = 45.5% SP = 43.8% For methylomics: SE = 31.4% SP = 52.4%
Margin Tree Classification	Tamaresis et al.^56^	All four stages of endometriosis^a^	Not specified	148 endometrial samples (77 endometriosis, 37 without endometriosis but other uterine/pelvic pathology, 34 controls)	FOSB, FOS, EGR1, JUNB, MTSS1L, CTSW, TGFB1, SOC3, IL32, FKBP8, ISYNA1, CCL3, GNLY, MAP3K11, C1QA, NOTCH3, CYR61, NPTXR, FBN1, PNRC2, ITGA6, DHFR, SLC39A6, MYO10, HSP90B1, SMC3, PKP4, PALLD, DIO2	SE = NR SP = NR

*NR* not reported, *SCAF11* SR-related CTD-associated factor 11, *KIF3A* kinesin family member 3A, *KRAS* Kirsten rat sarcoma viral oncogene homolog, *MDM2* mouse double minute 2 homolog, *FOSB* Fbj murine osteosarcoma oncogene B, *EGR1* early growth response 1, *JUNB* JunB proto-oncogene, *MTSS1L* metastasis suppressor 1-like, *CTSW* cathepsin W, *TGFB1* transforming growth factor beta 1, *SOC3* suppressor of cytokine signaling 3, *IL32* interleukin 32, *FKBP8* FKBP prolyl isomerase 8, *ISYNA1* inositol-3-phosphate synthase 1, *CCL3* chemokine ligand 3, *GNLY* granulysin, *MAP3K11* mitogen-activated protein kinase kinase kinase 11, *C1QA* complement C1q A chain, *NOTCH3* notch receptor 3, *CYR61* cysteine-rich angiogenic inducer 61, *NPTXR* neuronal pentraxin receptor, *FBN1* fibrillin 1, *PNRC2* protein rich nuclear receptor coactivator 2, *ITGA6* integrin subunit alpha 6, *DHFR* dihydrofolate reductase, *SLC39A6* Dolutegravir carrier family 39 member 6, *MYO10* myosin X, *HSP90B1* heat shock protein 90 beta family member 1, *SMC3* structural maintenance of chromosomes 3, *PKP4* plakophillin 4, *PALLD* Palladin, cytoskeletal associated protein, *DIO2* iodothyronine deiodinase 2, *SE* sensitivity, *SP* specificity.

^a^Minimal, mild, moderate and severe stages of endometriosis were included.

### Diagnostic or predictive models for endometriosis using mixed variables

Three studies^27,57,58^ used mixed variable types to create predictive or diagnostic models for endometriosis as shown in Table 6. All three studies used logistic regression as the methodology to construct models and the sample sizes ranged from 119 patients^57^ to 1396 patients^27^. Inputs included clinical variables collected from patient medical history, physical exam findings, ultrasonography evidence, and MRI visualization. It should be noted that Chattot et al.^57^ had the smallest sample size. The study with the largest sample size^27^ reported a SE and SP of 82.6% and 75.8%, respectively. The accuracy for studies in this category was relatively consistent compared to other categories with similar SE and SP.

**Table 6:** Diagnostic and predictive models built using mixed variables.

AI methods used	Authors [ref.]	Stage of endometriosis	Type of endometriosis	Sample size	Inputs used	Evaluation Metric
Logistic Regression	Guo et al.^58^	All stages of endometriosis and stage 3/4 endometriosis	NR	1016 infertile patients	for any-stage endometriosis nomogram: BMI, Cycle length, parity, palpable nodularity, endometrioma diagnosed on TVS, tubal pathology; for stage 3–4 endometriosis nomogram: pain, palpable nodularity, endometrioma diagnosed on TVS	SE = NR SP = NR
Logistic Regression	Chattot et al.^57^	Not specified	NR	119 patients (47 endometriosis with rectosigmoid involvement, 72 endometriosis without rectosigmoid involvement)	Palpation of a posterior nodule on digital examination, UBESS score of 3 on ultrasonography, rectosigmoid involvement in endometriosis infiltration on MRI, presence of blood in the stools during menstruation	SE = NR SP = NR
Logistic Regression	Nnoaham et al.^27^	Stage 3 and 4 endometriosis	NR	1396 symptomatic women	Ultrasound evidence, menstrual dyschezia, ethnicity, history of benign ovarian cysts	SE = 82.6% SP = 75.8%

*NR* not reported, *BMI* body mass index, *TVS* transvaginal ultrasound, *UBESS* ultrasound-based endometriosis staging system, *MRI* magnetic resonance imaging, *SE* sensitivity, *SP* specificity.

### Diagnostic or predictive models for endometriosis using imaging

Only three studies^59–61^ explored the use of imaging variables as their primary inputs for their AI models as seen in Table 7. Guerriero^59^ built models specifically for rectosigmoid endometriosis and compared the accuracy of the different AI methods using the same inputs for each model. This specific study allows one to draw conclusions about the accuracy of different methodologies in developing predictive models to increase suspicion for rectosigmoid endometriosis. The Naïve Bayes and SVM approaches produced the models with the highest accuracy (75%) in this study and K-nearest neighbor produced the lowest accuracy (69%). SVM also produced the highest SE at 84% while Naïve Bayes and decision tree showed the highest SP (77%). The pooled SE for the models with highest accuracy in each study was 88% while the pooled SP was 89.7%^59–61^.

**Table 7:** Diagnostic and predictive models built using imaging.

Authors [ref.]	Stage of endometriosis	Type of endometriosis	Sample size	Inputs used	AI methods used	Method accuracy
Maicus et al.^61^	NR	Endometriosis with POD obliteration	749 sliding sign transvaginal ultrasound videos	Presence of sliding sign on transvaginal U/S	Resnet (2 + 1)D	SE = 89% SP = 90%
Guerriero et al.^59^	NR	Rectosigmoid endometriosis	106 patients with U/S diagnosis of rectosigmoid endometriosis	Age; presence of U/S signs of uterine adenomyosis; presence of an endometrioma; adhesions of the ovary to the uterus; presence of “kissing ovaries”; absence of sliding sign	K-nearest Neighbor	SE = 66% SP = 71%
Guerriero et al.^59^	NR	Rectosigmoid endometriosis	106 patients with U/S diagnosis of rectosigmoid endometriosis	Age; presence of U/S signs of uterine adenomyosis; presence of an endometrioma; adhesions of the ovary to the uterus; presence of “kissing ovaries”; absence of sliding sign	Naive Bayes	SE = 72% SP = 77%
Guerriero et al.^59^	NR	Rectosigmoid endometriosis	106 patients with U/S diagnosis of rectosigmoid endometriosis	Age; presence of U/S signs of uterine adenomyosis; presence of an endometrioma; adhesions of the ovary to the uterus; presence of “kissing ovaries”; absence of sliding sign	Neural Networks	SE = 72% SP = 73%
Guerriero et al.^59^	NR	Rectosigmoid endometriosis	106 patients with U/S diagnosis of rectosigmoid endometriosis	Age; presence of U/S signs of uterine adenomyosis; presence of an endometrioma; adhesions of the ovary to the uterus; presence of “kissing ovaries”; absence of sliding sign	Support Vector Machine	SE = 84% SP = 71%
Guerriero et al.^59^	NR	Rectosigmoid endometriosis	106 patients with U/S diagnosis of rectosigmoid endometriosis	Age; presence of U/S signs of uterine adenomyosis; presence of an endometrioma; adhesions of the ovary to the uterus; presence of “kissing ovaries”; absence of sliding sign	Decision Tree	SE = 66% SP = 77%
Guerriero et al.^59^	NR	Rectosigmoid endometriosis	106 patients with U/S diagnosis of rectosigmoid endometriosis	Age; presence of U/S signs of uterine adenomyosis; presence of an endometrioma; adhesions of the ovary to the uterus; presence of “kissing ovaries”; absence of sliding sign	Random Forest	SE = 66% SP = 72%
Guerriero et al.^59^	NR	Rectosigmoid endometriosis	106 patients with U/S diagnosis of rectosigmoid endometriosis	Age; presence of U/S signs of uterine adenomyosis; presence of an endometrioma; adhesions of the ovary to the uterus; presence of “kissing ovaries”; absence of sliding sign	Logistic Regression	SE = 72% SP = 73%
Reid et al.^60^	NR	NR	189 women (100 training set, 89 test set) with suspected endometriosis	POD 1 model: posterior compartment deep endometriosis, right ovarian fixation, negative “sliding sign”; POD 2 model: unilateral ovarian fixation, unilateral endometrioma, negative “sliding sign”	Logistic Regression	POD 1: SE = 88% SP = 97% POD 2: SE = 88% SP = 99%

*U/S* ultrasound, *POD* pouch of Douglas, *NR* not reported, *SE* sensitivity, *SP* specificity.

Reid et al.^60^ also produced two logistic regression models using different imaging variables; the accuracy of both models was higher than the logistic regression model produced by Guerriero et al.^59^ indicating that perhaps the inputs for Reid’s model^60^ played a role in the higher accuracy, SE and SP. All three studies in this category explored “sliding sign” on transvaginal ultrasound as an important features in their models.

Maicus et al.^61^ was the only study to use a deep learning model called Resnet (2 + 1)D to classify the state of the pouch of Douglas with regards to adhesions indicative of endometriosis in patients. Their model was trained, internally validated, and externally tested on a dataset to evaluate the sliding sign on ultrasound, demonstrating an accuracy of 88.8%.

## Discussion

In the field of endometriosis, AI interventions have proven to be heterogenous in terms of their purpose, methodology, input selection and accuracy. Given the wide range of problems that exist in the field of endometriosis diagnosis, prediction and research, it is not surprising that models were built to tackle many different problem formulations. This study performed a thorough scoping review on the literature intersecting endometriosis and AI, and it provides a timely understanding of AI technology in the field of endometriosis. A meta-analysis of the data was not possible due to the diverse nature of studies included in this scoping review. Our study identified six major categories of model inputs that were used to build AI interventions in addition to three studies that used AI methods to improve research techniques^28–30^ and one study that only used lesion characteristics to build a predictive model^62^. Of the six major input categories, biomarkers, clinical variables, genetic variables and metabolite spectra were the most frequently used input types for building diagnostic and predictive AI models.

AI interventions that were built using biomarker inputs included diagnostic and predictive models for ultrasound-negative endometriosis^34^, and ovarian endometriomas^33^. Biomarker inputs for these models included plasma biomarkers collected in all phases of the menstrual cycle^34^, lipidomic profiling of endometrial fluid^33^, and serum miRNA markers^35^. AI interventions built using metabolite spectra as their primary input included detecting endometriosis in serum samples^43,44^, screening for biomarkers in eutopic endometrium^26^, diagnosing ultrasound-negative endometriosis^40^, diagnosing endometriosis using messenger RNA expression in endometrium biopsies^41^, identifying predictive serum biomarkers^42^, diagnosing and staging endometriosis using peptide profiling^39^, determining classifier metabolites for early prediction risk^38^, and diagnosing stage 3 and stage 4 endometriosis in infertile patients^36^. Studies that used genetic variables to build AI interventions included classifying endometriosis using RNAse and enrichment-based DNA-methylation datasets^53^, diagnosing endometriosis using gut and/or vaginal microbiome profiles^54^, using transcriptomics or methylomics to classify endometriosis^55^, and staging pelvic endometriosis using genomic data^56^. Some studies also used clinical signs and symptoms collected when obtaining a patient’s medical history as well as other clinical variables to build models. These AI interventions included predicting the presence of posterior deep endometriosis in patients with chronic pelvic pain symptoms^49^, predicting pregnancy rates in patients with endometriosis^48^, predicting medical care decision rules for patients with recurrent endometriomas^51^, diagnosing DE pre-operatively for patients with endometriomas^47^ and differentiating between patients with and without endometriosis^50^.

Our scoping review was able to evaluate the current literature and map out the field of study to demonstrate that AI applications in endometriosis look promising for improving diagnostics, research efficacy and outcome prediction in this patient population. Pooled SE ranged between 81.7 and 96.7% and pooled SP ranged between 70.7 and 91.6%. Our review included a range of heterogenous study designs, large retrospective analyses, various ML interventions and diverse research questions in the field of endometriosis. This is a timely review providing clinicians and computer scientists with an extensive understanding of AI applications in endometriosis. Clinical decision-making by humans is often prone to errors, biases and heuristics^63^. However, this review shows strong promise for AI’s ability to mitigate these human errors and provide superior outcome prediction with high SE and SP. Although many of the studies included in this review relied on a human component for data analysis/collection and determining feature extraction, AI technologies (especially when using standardized and validated models) may present the potential to reduce diagnostic error that can result from individual practicing biases and clinical heuristics. Future studies with human comparators are required to determine this. This review also demonstrated how AI can be used to improve research efficacy particularly through the use of natural language processing^28^ and identification of potential biomarkers^30^ and diseases^29^ associated with endometriosis pathophysiology. Lastly, this scoping review adds to future recommendations for research in this field and supports the need for standardized guidelines for ML applications in medicine.

Approximately 44.4% (*n* = 16) of AI interventions were predictive models meant to predict various outcomes in patients with endometriosis or undifferentiated symptomatic patients. Models were built to predict the presence of posterior DE in patients with chronic pelvic pain^49^, the clinical pregnancy rate in patients with endometriosis^48^, and many other outcomes in this patient population. However, many of these studies were conducted retrospectively and they did not adequately compare the AI’s ability to outperform existing decision tools and clinical diagnostics. Additionally, none of the studies involved a human comparator (since many models were trained and validated on retrospectively diagnosed patient datasets) and thus make it difficult to comment on AI’s superiority as a tool clinicians can use for predictive modeling.

The type and stage of endometriosis varied among the included studies; thus, the AI approaches to prediction and diagnosis also differed. This makes it difficult to compare AI models used in the studies. Many studies lacked detailed information on the methods used to verify patients with endometriosis with regards to a reference standard, while others cited gold standard laparoscopic visualization with subsequent histopathologic confirmation as the modality of diagnosis. Additionally, the heterogeneity of the study designs, input data used, and AI interventions, made it difficult to compare the accuracy and efficacy of the different models. Many studies lacked transparent descriptions of their modeling making it difficult to critique methodology and determine if the right AI model was being used to predict the outcome in question.

Applying AI to assess endometriosis is relatively new, and most AI methods used are still relatively simple. Various data types continue to be explored; however, each data type was utilized exclusively up to date. As can be seen from the tables, the use of protein spectra continues to be perhaps the most common approach, but generally only with small sample sizes. In the future, the increasing adoption of AI in assessing endometriosis will also likely play an essential role in women’s healthcare.

Our recommendations, based on this review and challenges of employing AI, are as follows:

1. The types and stages of endometriosis included in the study sample need to be clearly defined, and models should specify what type/stage of endometriosis they are built to predict, classify or diagnose.
2. The gold standard (a reference where we compare the AI model against) has to be defined and justified to assess reliability.
3. The evaluation metric (e.g., sensitivity and specificity) needs to be tested and reported clearly.
4. Transparent descriptions of the used AI model is needed for reproducibility.
5. Applying multiple AI models to determine the most accurate one for specific outcomes and diagnostic goals.
6. A large sample size with a diverse age group used is required for achieving generalizability.
7. Training and testing phases need to be clearly explained, specifically stating whether cross-validation or holdout is implemented; and
8. Logistic regression models incorporating a training and test/validation cohort would be more effective in establishing external validation of the model; and
9. Studies using retrospective analyses of large clinical datasets to build models should attempt to validate their models in prospective controlled clinical trials. Controlled clinical trials are required to determine whether AI can outperform human decision-making and remove any potential biases. Although internal validation samples are essential to test a model’s performance, these models should also be tested through prospective controlled trials to ensure that they are generalizable in a clinical context and that their performance is not limited to an artificial set of parameters.

Of the 36 studies included in this review, 50% were published in the last 5 years, indicating that there is recent and rapidly growing interest in AI applications to improve diagnostic, predictive and research capabilities for a complex disease such as endometriosis. Further research should be conducted using human comparators and should include comparisons with existing scoring systems and diagnostic tools to determine AI’s superiority for predictive and diagnostic modeling in endometriosis. These AI algorithms should also be externally validated or tested through prospective controlled trials to ensure that they contribute to advancing real-world clinical practice and diagnostics. This review was able to identify this interest in AI and highlight the benefits and shortcomings of AI interventions to improve future models for endometriosis.

## Methods

### Study guidelines

Given the heterogeneity and breadth of research in this field, a scoping review was performed to summarize the use of AI applications in endometriosis research, diagnostics, and prediction to help identify gaps in knowledge and address broad research questions^64^. The guidelines of the Preferred Reporting Items for Systematic Reviews and Meta-Analyses for Scoping Review (PRISMA-ScR)^65^ and Arksey and O’Malley’s recommendations for scoping review methodology^66^ were followed. A prior review protocol was drafted using the Preferred Reporting Items for Systematic Reviews and Meta-Analyses Protocols^67^ for internal use amongst the research team but it was not externally published or registered prospectively.

### Search strategy and study eligibility

The PubMed, Medline-OVID, EMBASE, and CINAHL databases were searched sequentially from January 2000 to March 2022 for all English-language papers using the following search strategy (adapted for each database): [(Endometriosis) OR (Endometrioma)] AND [(AI) OR (ML) OR (Prediction Model) OR (Classification)]. Gray literature was not included in this scoping review in attempt to only include peer-reviewed studies. This timeframe was chosen to reflect advances in AI technologies and applications in medicine. The scope of the search was not restricted to a particular type or stage of endometriosis. The search for this scoping review was completed in March 2022.

### Inclusion and exclusion criteria

The following inclusion criteria were used to determine study eligibility for this review: (1) the study involved assessing an AI approach or model to advance prediction, diagnosis, management or disease understanding in the field of endometriosis; (2) the study reported a quantitative metric on the accuracy/performance of the AI method; (3) the study was conducted using humans; (4) the article was accessible in English; and (5) the study used a validation method to test its model. Studies were excluded if: (1) they were not conducted using humans; (2) did not assess or evaluate an AI approach or model; (3) did not pertain to the field of endometriosis; and (4) developed a logistic regression model without the use of a training and test/validation set. One reviewer (BS) conducted the literature search and two reviewers (BS and ME) screened the titles, abstracts and full-texts independently for potentially eligible studies. Reference lists of eligible studies were also hand-searched but no additional studies were included on this basis.

### Study selection and data extraction

One author (B.S.) conducted the literature search, and two authors (B.S. and M.E.) independently screened the titles and abstracts for potentially eligible studies. Each potential study for inclusion underwent full-text screening and was assessed to extract study-specific information and data; Table 1 presents a summary of the title, lead author, publication year, study design, AI intervention, purpose/aim, sample size, type of inputs used in the AI method, specific inputs in the final model, evaluation metrics used and AI accuracy. Two reviewers (B.S. and M.E.) independently conducted a full-text screening and extracted information from potentially eligible studies. They then cross-checked the identified studies to determine eligibility through discussion and used consensus to resolve discrepancies. The information collated in the initial evidence table was used to aggregate data and determine the main themes of use for AI in endometriosis in the currently published literature. Where studies explored more than one AI model, the model with the highest accuracy was assessed and included in the review.

### Pooled evaluation metric

Pooled sensitivities and specificities were calculated for studies within the same input category. The following formula^68^ was used to combine means across different studies where SE or SP is the pooled mean for sensitivity or specificity, as follows:

1 $${{{\mathrm{SE}}}}\,{{{\mathrm{or}}}}\,{{{\mathrm{SP}}}} \,=\, \frac{{N_1X_1 \,+\, N_2X_2 \,+\, \cdots }}{{N_1 \,+\, N_2 \,+\, \cdots }}$$

where, for example, *N*_1_ is the number of participants in study 1 and *X*_1_ is the value of the reported sensitivity or specificity in study 1.

## Data Availability

The authors declare that all data supporting the findings of this study are available within the paper.
